# RETRACTION: Anemia and Contributing Factors in Severely Malnourished Infants and Children Aged between 0 and 59 Months Admitted to the Treatment Centers of the Amhara Region, Ethiopia: A Multicenter Chart Review Study

**DOI:** 10.1155/anem/9874051

**Published:** 2025-10-07

**Authors:** Anemia

RETRACTION: W. W. Takele, A. G. Baraki, H. F. Wolde, et al., “Anemia and Contributing Factors in Severely Malnourished Infants and Children Aged between 0 and 59 Months Admitted to the Treatment Centers of the Amhara Region, Ethiopia: A Multicenter Chart Review Study,” *Anemia* 2021 (2021): 6636043, https://doi.org/10.1155/2021/6636043.

The above article, published online on 28 March 2021 in Wiley Online Library (https://wileyonlinelibrary.com), has been retracted following the agreement of the journal's Academic Editor, Shamreen Naaz; and John Wiley & Sons Ltd.

The retraction has been agreed following an investigation by the publisher, which identified substantial overlap with an article published earlier by the same authors [1].

The authors have been informed of this retraction but did not provide a response.
